# Studying the trafficking of labeled trodusquemine and its application as nerve marker for light‐sheet and expansion microscopy

**DOI:** 10.1096/fj.202201276R

**Published:** 2022-11-24

**Authors:** Claudia Capitini, Luca Pesce, Giulia Fani, Giacomo Mazzamuto, Massimo Genovese, Alessandra Franceschini, Paolo Paoli, Giuseppe Pieraccini, Michael Zasloff, Fabrizio Chiti, Francesco S. Pavone, Martino Calamai

**Affiliations:** ^1^ European Laboratory for Non‐Linear Spectroscopy (LENS) University of Florence Sesto Fiorentino Italy; ^2^ Department of Physics University of Florence Sesto Fiorentino Italy; ^3^ Department of Experimental and Clinical Biomedical Sciences, Section of Biochemistry University of Florence Florence Italy; ^4^ National Institute of Optics – National Research Council (CNR‐INO) Sesto Fiorentino Italy; ^5^ CISM – Mass Spectrometry Centre University of Florence Sesto Fiorentino Italy; ^6^ Enterin Inc. Philadelphia Pennsylvania USA; ^7^ MedStar‐Georgetown Transplant Institute Georgetown University School of Medicine Washington DC USA

**Keywords:** intracellular trafficking, lipid membrane, nerve fiber staining, neurodegeneration, optical imaging, squalamine

## Abstract

Trodusquemine is an aminosterol with a variety of biological and pharmacological functions, such as acting as an antimicrobial, stimulating body weight loss and interfering with the toxicity of proteins involved in the development of Alzheimer's and Parkinson's diseases. The mechanisms of interaction of aminosterols with cells are, however, still largely uncharacterized. Here, by using fluorescently labeled trodusquemine (TRO‐A594 and TRO‐ATTO565), we show that trodusquemine binds initially to the plasma membrane of living cells, that the binding affinity is dependent on cholesterol, and that trodusquemine is then internalized and mainly targeted to lysosomes after internalization. We also found that TRO‐A594 is able to strongly and selectively bind to myelinated fibers in fixed mouse brain slices, and that it is a marker compatible with tissue clearing and light‐sheet fluorescence microscopy or expansion microscopy. In conclusion, this work contributes to further characterize the biology of aminosterols and provides a new tool for nerve labeling suitable for the most advanced microscopy techniques.

**Abbreviations**
4‐HT: 4‐hydroxy‐TEMPO
AcX: 6‐([acryloyl]amino)hexanoic acid, Succinimidyl Ester
APS: ammonium persulfate
BSA: bovine serum albumin
DMSO: dimethyl sulfoxide
EF: expansion factor
ExM: expansion microscopy
GA: glutaraldehyde
LUVs: largeunilamellar vesicles
LSFM: light‐sheet fluorescence microscopy
MA‐NHS: methacrylic acid N‐hydroxysuccinimide Ester
MBP: myelin basic protein
PBS‐T: PBS‐Triton X‐100
PFA: paraformaldehyde
PTP1B: protein‐tyrosine phosphatase1B
ROS: reactive oxygen species
RT: room temperature
SD: standard deviation
SIM: simvastatin
TDE: 2,2′‐thiodiethanol
TEMED: tetramethylene diamine
TRO‐A594: trodusqueminecoupled with Alexa Fluor® 594 Succinimidyl Ester
TRO‐ATTO565: trodusquemine coupledwith ATTO 565 Succinimidyl Ester

## 1 INTRODUCTION

Aminosterols represent a class of natural compounds isolated from the liver of the dogfish shark Squalus acanthias.^1^, ^2^ Among those, the first to be isolated has been squalamine; this small molecule was found to exhibit bactericidal activity against Gram‐negative and Gram‐positive bacteria,^1^, ^3^ as well as antiangiogenic properties against retinopathy and cancer^4^, ^5^, ^6^ and antiviral activity.^7^ An additional aminosterol that has attracted a great deal of interest is trodusquemine (also known as MSI‐1436), which, similarly to squalamine, was shown to have antimicrobial activity, with an efficacy even greater than that of squalamine and against a large variety of microorganisms.^2^ Moreover, trodusquemine was shown to suppress appetite and decrease body weight in a highly specific manner in normal and obese rodents,^8^, ^9^ by acting specifically and reversibly as a non‐competitive inhibitor of the protein‐tyrosine phosphatase 1B (PTP1B), a negative regulator of insulin and leptin signaling that represents a validated therapeutic target for diabetes and obesity.^10^, ^11^ The finding that trodusquemine determines an enhancement of insulin‐stimulated tyrosine phosphorylation of hypothalamic insulin receptor β in a mouse model of diet‐induced obesity proves that trodusquemine is able to cross the blood–brain barrier.^10^

Of particular significance in the context of neurodegeneration, squalamine and trodusquemine were recently found to affect the aggregation process of α‐synuclein and Aβ,^12^, ^13^, ^14^ which are associated with Parkinson's^15^, ^16^, ^17^ and Alzheimer's diseases,^17^, ^18^, ^19^, ^20^, ^21^ respectively. They were also found to suppress the cytotoxicity caused by oligomers formed from the same protein systems, through a similar mechanism consisting in the displacement of toxic oligomers from cell membranes,^12^, ^13^, ^14^, ^22^, ^23^ even if trodusquemine was found to be effective at lower doses with respect to squalamine.^22^, ^23^

Although the protective role of aminosterols against α‐synuclein and Aβ misfolded protein oligomers has been determined with findings obtained both in vitro and in vivo, only recently we have reported a detailed characterization of the binding ability of trodusquemine to membrane lipids, and of its impact on the physicochemical properties of lipid membranes.^24^ This study was prevalently carried out using large unilamellar vesicles (LUVs), formed by four lipids normally found in neuronal membranes to model membrane lipid bilayers, formed in the presence of trodusquemine. Confocal microscopy images, FRET analysis, and NMR spectra revealed a strong interaction between LUVs and trodusquemine. The binding was also observed between fluorescently labeled trodusquemine and the cell membrane of human neuroblastoma cells.^24^ In particular, FRET data showed that trodusquemine coupled to BODIPY dye preferentially interacted with cholesterol of LUVs and, to a lower extent, with the ganglioside GM1, relative to phosphatidylcholine and sphingomyelin.^24^ Binding of unlabelled trodusquemine to LUVs resulted in physicochemical changes in the lipid bilayer, which were expected to make the membrane more resistant to the action of misfolded oligomers.^24^ In fact, trodusquemine was also found to reduce the binding affinity of toxic oligomers formed from the HypF‐N protein for LUV membranes.^25^ Very little is known, however, on the binding properties of trodusquemine, and of aminosterols more in general, to the plasma membrane of living cells and their cellular routes of trafficking.

Here, we describe the processes that follow the initial interaction of trodusquemine with the plasma membrane of living neuroblastoma cells. To this aim, we used trodusquemine labeled with Alexa Fluor® 594 (termed TRO‐A594), or alternatively with ATTO 565 (termed TRO‐ATTO565). The difference between the two dyes is that Alexa Fluor® 594 is hydrophilic and negatively charged, whereas ATTO 565 is neutral and moderately hydrophilic and does not alter, therefore, the positive net charge of trodusquemine. These dyes constitute a better alternative to neutral and hydrophobic dyes such as BODIPY TMR‐X, which can already bind per se, to a certain extent, to the plasma membrane. The results show that trodusquemine binds to the plasma membrane independently of the covalently linked dye and, when internalized, is mainly targeted to lysosomes. We also demonstrated that TRO‐A594 displays a lower affinity to the plasma membrane of cholesterol‐depleted cells. Moreover, considering that trodusquemine is able to pass the blood–brain barrier and that myelin is enriched in cholesterol, we explored the binding properties of TRO‐A594 to neuronal tissue and discovered a striking high affinity to neuronal fibers, by performing mesoscopic reconstruction of mouse brain slices using the clearing agent 2,2′‐thiodiethanol (TDE)^26^, ^27^ coupled with light‐sheet fluorescence microscopy (LSFM) and expansion microscopy (ExM).^28^ Analogue results were found when brain slices were subjected to expansion. This work addresses both biological and methodological aspects linked to trodusquemine, ranging from the study of its potential fate following the primary interaction with the cell membrane, to its characterization as a novel and versatile tool to specifically stain nerve fibers for a plethora of biological applications, from nerve tracing to histopathological investigations.

## 2 MATERIALS AND METHODS

### 2.1 Trodusquemine labeling

Trodusquemine was synthesized as previously described^8^ as a hydrochloride salt, at a purity higher than 97% as measured by mass spectrometry. Trodusquemine was dissolved in 0.1 M sodium bicarbonate buffer, pH 7.0 to obtain a 10 mM stock solution and stored at 4°C. The Alexa Fluor® 594 Succinimidyl Ester dye (Thermo Fisher Scientific, Waltham, MA, USA) was dissolved in anhydrous dimethyl sulfoxide (DMSO) to obtain a 15 mM stock solution and stored at −20°C. The conditions of the reaction were 5 mM trodusquemine, 0.5 mM dye, in a final 100 μl‐volume of 0.1 M sodium bicarbonate buffer, pH 7.0 at 25°C for 1 h under mild orbital shaking in the dark, in order to obtain a molar ratio of 1:10 between the fluorescent dye and trodusquemine (Figure S1A–C). In these reaction conditions, the aminosterol carries only one fluorescent dye at the level of its primary amine, and no unreacted dye is detected with mass spectrometry, whereas both unlabeled and labeled trodusquemine were detected with the expected molecular weights of 683.60 g/mol and 1307.60 g/mol, respectively (Figure S1D). We refer to labeled‐trodusquemine as TRO‐A594.

For the labeling with ATTO 565 NHS ester (Sigma‐Aldrich), the dye was dissolved in DMSO to obtain a 28 mM stock solution and stored at −20°C. The labeling solution was prepared following the manufacturer's instructions: 1 volume of 0.2 M sodium bicarbonate buffer, pH 9.0, was added to 20 volumes of PBS buffer, to obtain the labeling buffer at pH 8.3. The reaction conditions were 5 mM trodusquemine, 0.5 mM dye, in a final 20 μl‐volume of labeling solution, at 25°C for 18 h under mild orbital shaking in the dark, in order to obtain a molar ratio of 1:10 between the fluorescent dye and trodusquemine (Figure S2). We refer to labeled‐trodusquemine as TRO‐ATTO565.

### 2.2 Neuroblastoma cell cultures

Human SH‐SY5Y neuroblastoma cells (A.T.C.C. Manassas, VA, USA) were cultured in Dulbecco's Modified Eagle's Medium (DMEM, ThermoFisher Scientific, Waltham, MA, USA) F‐12 supplemented with 10% FBS, 1 mM glutamine, and 1% penicillin/streptomycin solution. Cell cultures were kept in a humidified atmosphere at 37°C and 5% CO_2_, and grown until they reached 90% confluence.

### 2.3 Enzymatic assay

Recombinant human PTP1B_1–302_ was expressed as a hexahistidine tagged (His6) fusion protein in BL21 *E. coli* cells and purified by affinity chromatography using a column loaded with a nickel iminodiacetic acid resin. Enzymatic assays were performed in 96‐well plates diluting an aliquot of enzyme (50 nM) in the assay buffer containing 0.075 M β‐β‐dimethylglutarate pH 7.0, 1 mM EDTA, 0.1 mM dithiothreitol and 2.5 mM of substrate (*p*‐nitrophenyl phosphate, *p*NPP). After 20 min, the reactions were stopped adding 100 μl of 0.2 M KOH in each plate. Then, the amount of *p*‐nitrophenol released was obtained by measuring the absorbance of samples at 405 nm using a Synergy H1 microplate reader (BioTek Instruments, Winooski, Vermont, U.S.A). The IC_50_ (the concentration of the inhibitor at which the enzymatic activity is decreased of up to 50%) values were determined fitting experimental data to the following equation:

$$\frac{V_{i}}{V_{0}}=\frac{\mathrm{Max}-\mathrm{Min}}{1+{\left(\frac{x}{{\mathrm{IC}}_{50}}\right)}^{\text{slope}}}+\mathrm{Min}$$

by nonlinear regression analysis using OriginPro 2021 (OriginLab Corporation, Northampton, MA, USA). In the above equation, *V*_*i*_ */V*_*0*_ represents the relative activity obtained in presence of each inhibitor concentration, *Max* and *Min* represent the maximum and minimum values of the activity, respectively, *x* is the concentration of the inhibitor, and *slope* represents the slope of the curve in the transition zone.^29^

### 2.4 Treatment of neuroblastoma cells with trodusquemine and confocal microscopy analysis

SH‐SY5Y cells were plated on glass coverslips in 12‐well plates at 100 000 cells/well density. Twenty‐four hours after plating, the cells were washed with PBS and incubated with 5 μM TRO‐A594 for different times. Ten minutes before the end of each incubation time, the Hoechst 33342 dye was added to the culture medium at a concentration of 10 μg/ml in order to stain the nuclei of the cells. After washing with PBS, the incubation medium was replaced with the Leibovitz's L‐15 (Thermo Fisher Scientific), a medium designed for supporting cell growth in the absence of CO_2_ equilibration. In the pulse‐chase experiment, TRO‐A594 at the same concentration of 5 μM was added to the cell culture medium and after 15 min incubation was removed by replacing the medium with fresh one. The uptake of trodusquemine was then tracked after 1, 2, 3, 24, and 48 h. The analysis of TRO‐A594 and nuclei‐derived fluorescence intensities was performed after excitation at 561 and 405 nm, respectively, using a Nikon C2 laser scanning confocal microscope and a Plan Fluor 100× 1.49 NA oil immersion objective. Optical sections at median planes of the cells (1024 × 1024 pixels) were taken for each sample and analyzed using the Fiji software. All settings, that is, laser power, detector gain, and pinhole diameter, were kept constant for each analysis. Images were acquired using sub‐saturation settings.

Membrane‐bound TRO‐A594 levels were assessed by selecting multiple ROIs per cell along the plasma membrane, while the cytoplasmic trodusquemine levels were measured by selecting multiple ROIs per cell in the cytoplasmic compartment, excluding the nucleus area, after background subtraction. We used manual segmentation in place of automatic, as the standard plasma membrane marker to make the binary mask for segmentation (fluorescently labeled wheat germ agglutinin) is not exclusively found on the plasma membrane but goes also in the cytoplasm, making very inaccurate the automatic selection of the localization of trodusquemine. The same experiment was also carried out with TRO‐ATTO565.

### 2.5 Measurement of cytosolic free Ca^2+^ levels and intracellular ROS production

The cytosolic Ca^2+^ levels and the ROS production were measured in living SH‐SY5Y cells plated in 12‐well plates containing coverslips at the density of 150 000 cells/well. After 24 h the cells were washed with PBS and incubated with no treatment with 1 μM ionomycin for 1 h for the Ca^2+^ level detection or 250 μM H_2_O_2_ for 1 h for the ROS production assay, and with 5 μM trodusquemine for different times. Cells were then loaded with 4 μM Fluo‐4 AM (Thermo Fisher Scientific) for 10 min after the treatments, to detect the Ca^2+^ ions, or with 5 μM CM‐H_2_DCFDA (Thermo Fisher Scientific) the last 15 min of treatments, to detect ROS. Cells were analyzed using a TCS SP8 scanning confocal microscopy system equipped with an argon laser source (Leica Microsystems, Mannheim, Germany), after excitation at 488 nm. A series of 1‐μm‐thick optical sections (1024 × 1024 pixels) was taken using a Leica Plan Apo 63× oil immersion objective and projected along the z‐axis as a single composite image. >10 cells, in three different experiments, were analyzed using ImageJ software.

### 2.6 Live cell imaging for colocalization analysis with mitochondria and lysosomes

SH‐SY5Y cells were plated in 12‐well plates containing glass coverslips at 100 000 cells/well density. Twenty‐four hours after plating, the cells were washed with PBS and incubated with 5 μM TRO‐A594 and imaged at 2 and 24 h. For the study of colocalization with mitochondria, cells were washed with PBS and treated with pre‐heated fresh medium containing the MitoTracker™ Green FM (Thermo Fisher Scientific) solution in order to get a final probe concentration of 200 nM. After 15 min of incubation, cells were washed one time with Leibovitz's L‐15 medium and analyzed by confocal microscopy. The TRO‐A594 and mitochondria‐derived fluorescence values were analyzed after excitation at 561 and 488 nm, respectively. For the colocalization analysis, a Plan Fluor 100× 1.49 NA oil immersion objective was used, and a series of 1 μm thick optical sections (1024 × 1024 pixels) through the cell depth for each sample was taken.

In order to properly interpret the confocal images and quantify the degree of colocalization, the JACoP plug‐in^30^ available under the Fiji software was used, in particular, to determine the Pearson's correlation coefficient,^31^ an estimate of the covariance in the signal levels of two images, analyzing them pixel‐by‐pixel, and the Manders' overlap coefficient,^32^ an indicator of pixel fraction of the image 1 that overlaps with image 2 and vice versa.

For the study of colocalization with lysosomes, cells incubated for 2 h with 5 μM TRO‐ATTO565, were treated with medium containing 1X LysoView™ 488 (Biotium, Fremont, CA, USA) for 30 min at 37°C before confocal acquisition. The TRO‐ATTO565 and lysosomes‐derived fluorescence values were analyzed after excitation at 561 and 488 nm, respectively.

### 2.7 Transient transfection for colocalization analysis with early endosomes, Golgi apparatus, and lysosomes

The plasmids named GFP‐EEA1 wt (Addgene, plasmid #42307),^33^ LAMP1‐mGFP (Addgene, plasmid #34831),^34^ and mEmerald‐TGNP‐N‐10 (Addgene, plasmid #54279) were used for the overexpression in SH‐SY5Y cells of early endosomes, lysosomes, and Golgi apparatus markers, respectively. SH‐SY5Y cells were plated in 12‐well plates containing glass coverslips at 100 000 cells/well density. Twenty‐four hours after plating, the cells were washed with PBS and separately transfected using FuGENE® HD Transfection Reagent (Promega Corporation, Madison, WI, USA), according to the manufacturer's instructions, with 1 μg of plasmid, 3 μl of FuGENE® HD in Opti‐MEM (ThermoFisher Scientific) for 24 h in a 5% CO_2_ humidified atmosphere at 37°C. Five μM of TRO‐A594 was added for 15 min to the transfected cells 2 and 24 h before the confocal microscopy experiments. After incubation, living cells were washed with PBS and maintained in Leibovitz's L‐15 medium. The TRO‐A594 and protein marker‐derived fluorescence intensities were analyzed as described in the previous subsection. A similar experiment was also performed incubating cells, previously transfected with the LAMP1‐mGFP plasmid, for 2 h with 5 μM of TRO‐ATTO565. As a positive control, cells transfected with GFP‐EEA1 wt were fixed in 4% (w/v) buffered paraformaldehyde (PFA) in PBS for 10 min at room temperature (RT), washed three times 5 min each in PBS, and then permeabilized with 0.1% (v/v) Triton X‐100 for 10 min at RT. After three washes of 5 min each in PBS at RT, the fixed sample was blocked with 4% BSA in PBS for 1 h. Cells were then incubated with 1:1000 rabbit polyclonal anti‐Rab5 antibody (Abcam) with 4% BSA in PBS overnight at 4°C. After 3 washes of 10 min each in PBS at RT, the specimen was incubated with 1:500 Alexa Fluor 488‐conjugated secondary antibody (ThermoFisher Scientific) with 4% BSA in PBS for 45 min at room temperature. Finally, the sample was washed three times with PBS, 10 min each and then stained with DAPI at the concentration of 5 μg/ml. The confocal microscopy experiment was then performed as described above.

### 2.8 Cholesterol depletion

Cholesterol was depleted from SH‐SY5Y cells by supplementing the culture medium with or without Simvastatin at 10 μM concentration for 48 h at 37°C. Fifteen minutes before confocal acquisition, cells were washed with PBS and treated with 5 μM TRO‐A594. The Hoechst 33342 dye was added to the culture medium to stain the nuclei of the cells, as reported above.

### 2.9 Mouse brain

Adult male and female C57BL/6 mice (age between 3 and 6 months) were used. They were housed in groups of a maximum of four animals. Mice received food and water ad libitum and were kept in a room under controlled light and dark cycle (12/12 h; light starts at 7:00 AM), temperature (22 ± 2°C), and humidity (55 ± 10%). All experimental procedures were approved by the Italian Ministry of Health (Authorization n. 512‐2018_FC). All experiments were conducted according to principles of the 3Rs. For the brain extraction, animals were deeply anesthetized with isoflurane (1.5–2%) and then transcardially perfused with 50 ml of ice‐cold 0.01 M PBS solution (pH 7.4), followed by 75 ml of 4% PFA. After perfusion, the brains were dissected out and further fixed in 4% PFA in PBS at 4°C for 24 h. The brains were then washed with PBS and embedded in 4% agarose for cutting; slices of 100 μm thickness were generated by using a vibratome (LEICA VT1000 S) and stored in PBS at 4°C. For the TRO‐A594 treatment, mouse brain slices were incubated with 5 μM TRO‐A594 for 15 min, 2, and 24 h, followed by three washes with PBS and then stained with DAPI at the final concentration of 5 μg/ml. Separately, mouse brain slices were also incubated with 5 μM TRO‐ATTO565 for 2 h. Stained mouse slices were acquired using confocal microscopy by exciting the samples at 561 and 405 nm, for TRO‐A594 (or TRO‐ATTO565) and nuclei‐derived fluorescences, respectively, using a Plan Fluor 100× 1.49 NA oil immersion objective. TRO‐A594 fluorescence was analyzed using the ImageJ software.

In another experiment, mouse slices were blocked with 1% BSA in PBS‐Triton X‐100 (PBS‐T, with 1% Triton X) for 2 h and washed three times for 45 min each with PBS‐T. Then, the slices were simultaneously stained with 5 μg/ml TRO‐ATTO565 and immunolabeled with 1:1000 Myelin Basic Protein (MBP, ARG23031, Arigobio), both in PBS‐T, overnight. After a 3 h‐washing with PBS‐T slices were incubated with 1:500 secondary Alexa 488 antibody (Thermo Fisher Scientific) in PBS‐T for 2 h, then were washed 3 times with PBS‐T for 45 min each and analyzed on confocal microscope by exciting at 561 and 488 nm, for TRO‐ATTO565 and MBP fluorescences, respectively.

For the ExM experiments, after 24 h of incubation with TRO‐A594, the mouse brain slices were incubated with 0.1 mg/ml AcX for ~16 h at 4°C. After two washes of 15 min each on ice, the treated samples were incubated in the hydrogel solution supplemented with 0.01% (w/w) 4‐hydroxy‐TEMPO (4‐HT), 0.2% (w/w) tetramethylene diamine (TEMED) and 0.2% (w/w) ammonium persulfate (APS), with the initiator APS added last, for 30 min at 4°C and then for ~2 h at 37°C (4‐HT and the incubation at 4°C was performed to prevent premature gelation). After gelation, overnight digestion in the digestion buffer was performed to obtain an isotropic expansion of the tissue. Next, the digested tissues were stained with DAPI at the final concentration of 5 μg/ml. Finally, incubation in distilled water was carried out for a complete expansion (2 h, water exchange every 30 min). The expanded tissue was acquired using confocal microscopy, with a Plan Fluor 20×, 0.9 NA, WD 1 mm water immersion objective lens. The expansion factor was calculated using the Fiji software by measuring the length of the pre‐expansion and post‐expansion hydrogels (*n* = 3).

### 2.10 LSFM of mouse brain

For the LSFM acquisition, mouse brain slices were incubated with 5 μM TRO‐A594 for 24 h, and equilibrated in 30% (w/w) and 68% (w/w) TDE for 1 h, to match the refractive index to 1.46 and make the tissue completely transparent. The whole slice acquisition was performed using a custom‐made, dual‐view, inverted light sheet fluorescence microscope with two 12×, NA 0.53, WD 10 mm excitation and emission objectives which are tilted at 45° relative to the sample plane. The whole system is controlled with a custom data acquisition and control software specifically developed for this setup, as reported in Pesce et al.^35^

### 2.11 ExM of cultured neuroblastoma cells

For the ExM experiments, SH‐SY5Y cells were plated in 12‐well plates containing glass coverslips at 250 000 cells/well density. Twenty‐four hour after plating, the cells were washed with PBS and incubated with 5 μM TRO‐A594 for 15 min. Cells were then washed three times with PBS and incubated in a complete culture medium at 37°C for 24 h. Next, the specimens were fixed with 4% PFA and 0.2% glutaraldehyde (GA) for 10 min on ice. After three washes of 5 min each on ice, the samples were incubated with 0.1 mg/ml 6‐([acryloyl]amino)hexanoic Acid, Succinimidyl Ester (AcX, Thermo Fisher Scientific, A20770)^36^ for 2 h at RT,^37^ or 25 mM methacrylic acid N‐hydroxysuccinimide ester (MA‐NHS; 730 300)^38^, ^39^ for 1 h at RT. After two washes with PBS for 15 min each, the functionalized samples were incubated in the hydrogel solution consisting of 2 M NaCl, 2.5% (w/w) acrylamide, 0.15% (w/w) N,N′‐methylenebisacrylamide, 8.625% (w/w) sodium acrylate, 0.2% (w/w) TEMED and 0.2% (w/w) APS in distilled water, with the initiator APS added last, for ~30 min at 37°C. The mixed gelling solution was kept in an ice bath to prevent the premature gelation. After gelation, the samples were soaked in the digestion solution consisting of 50 mM Tris‐HCl (pH 8), 1 mM EDTA, 0.5% Triton X‐100, 1 M NaCl, supplemented with 8 unit ml^−1^ proteinase K added freshly for ~2 h at 37°C, or 45 min at 37°C for the transfected samples.^36^, ^37^ Following the digestion step, the samples were extensively washed with PBS at RT and incubated with the DAPI dye at a final concentration of 5 μg/ml in order to stain the nuclei of the cells. Then, the samples were incubated in distilled water for 2 h, exchanging water every 30 min, to achieve the full expansion. The expansion factor (EF) was calculated using the Fiji software by measuring the area of the pre‐expansion, post‐digestion, and post‐expansion hydrogels (*n* = 4). The TRO‐A594 and nuclei‐derived fluorescences were acquired on confocal microscope after excitation at 561 and 405 nm, respectively, using a Plan Fluor 100× 1.49 NA oil immersion objective, and a series of 1‐μm‐thick optical sections (1024 × 1024 pixels) through the cell depth for each sample was taken. The pinhole size for pre‐ and post‐digestion samples acquisition was kept at 60 μm, while for the post‐expansion specimens acquisition was tuned at 150 μm to image the entire TRO‐A594 particle diameter. The analysis of the particle size distribution of TRO‐A594 vesicles in living, fixed (pre‐digestion), post‐digestion, and post‐expansion samples was performed using the Fiji software by measuring the area of single vesicles selected by ROIs and normalizing the values obtained to the EF.

### 2.12 Statistical analysis

With the exception of Figure 4, data are expressed as mean ± standard deviation (SD); when needed, statistical significance is evaluated using Student's *t* test. Statistical analysis was performed using the software KaleidaGraph.

## 3 RESULTS

### 3.1 TRO‐A594 accumulates in the cytoplasm over time

In order to demonstrate that trodusquemine has an affinity for the lipid bilayer, it was recently shown that TRO‐A594 binds to the plasma membrane of SH‐SY5Y neuroblastoma cells after 15 min incubation, with only a low amount localizing in the cytoplasm.^24^ Here, we extended our analysis to longer incubation times to investigate variations in localization.

**FIGURE 1:**
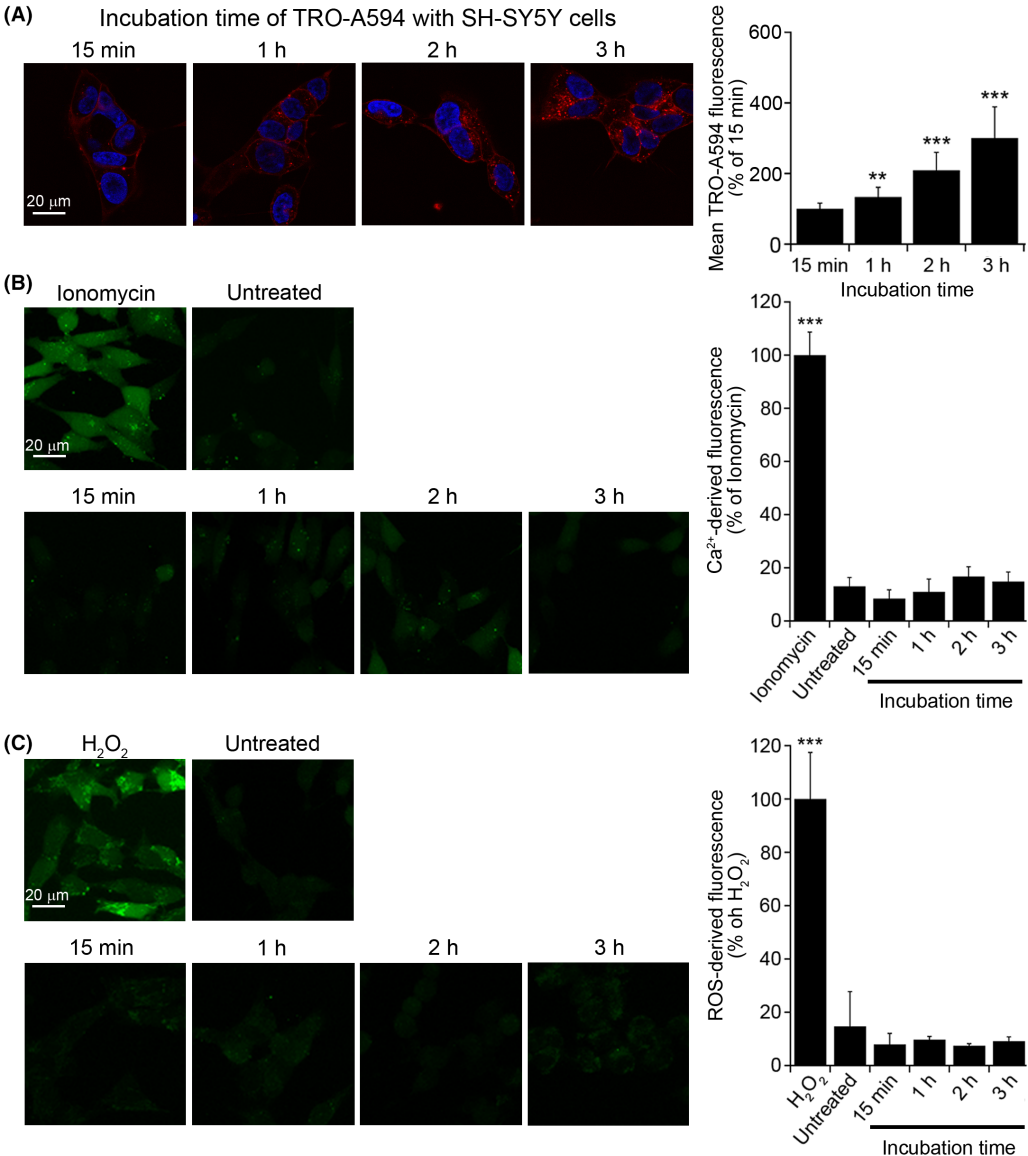
TRO‐A594 accumulation in SH‐SY5Y neuroblastoma cells over time. (A) Representative confocal scanning microscope images of SH‐SY5Y cells incubated for different times with 5 μM TRO‐A594. Red, TRO‐A594; blue, Hoechst. The images were analyzed at median planes parallel to the coverslip. *n* > 50 cells, from three independent experiments; error bars, SD; Student's *t*‐test, **p < .01; ***p < .001 with respect to the immediately preceding incubation time. (B and C) Representative confocal images of SH‐SY5Y cells showing (B) cytosolic free Ca^2+^ (green) and (C) intracellular ROS levels (green) after incubation for different times with 5 μM trodusquemine. Ionomycin and H_2_O_2_ were used as a positive control for Ca^2+^ and ROS analysis, respectively. *n* > 50 cells, from three independent experiments; error bars, SD; Bonferroni test, ***p < .001 with respect to (B) ionomycin and (C) H_2_O_2_.

TRO‐A594 was added at a concentration of 5 μM to the cell culture medium of human neuroblastoma SH‐SY5Y cells, and the cells were then analyzed by confocal microscopy after 15 min, 1, 2, and 3 h of treatment (Figure 1A). In agreement with previous results,^24^ after 15 min incubation TRO‐A594 localizes mostly on the plasma membrane. The inability of the dye to bind per se to the membrane was previously ascertained.^24^ At higher incubation times (1, 2, and 3 h), the TRO‐A594 fluorescence was found to significantly increase with time due to its persistent membrane localization and gradual accumulation into the cytoplasm (Figure 1A). These results suggest that trodusquemine not only is able to interact with the cell membrane, but it also accumulates into the cytoplasm at longer times.

To assess whether the increasing cytoplasmic accumulation of TRO‐A594 caused cellular dysfunction, potential changes in levels of intracellular Ca^2+^ (Figure 1B) and reactive oxygen species (ROS) (Figure 1C) were measured in SH‐SY5Y cells treated with the aminosterol under the same conditions mentioned above. The results showed that the increasing intracellular levels of trodusquemine did not induce deleterious effects on cell viability, as indicated by the Ca^2+^‐derived and ROS‐derived fluorescence intensities, which remained comparable to the corresponding values observed for the untreated cells. Moreover, the presence of the dye did not decrease the capability of trodusquemine to inhibit PTP1B activity in vitro (Figure S3).

### 3.2 TRO‐A594 is rapidly internalized in the cytoplasm

**FIGURE 2:**
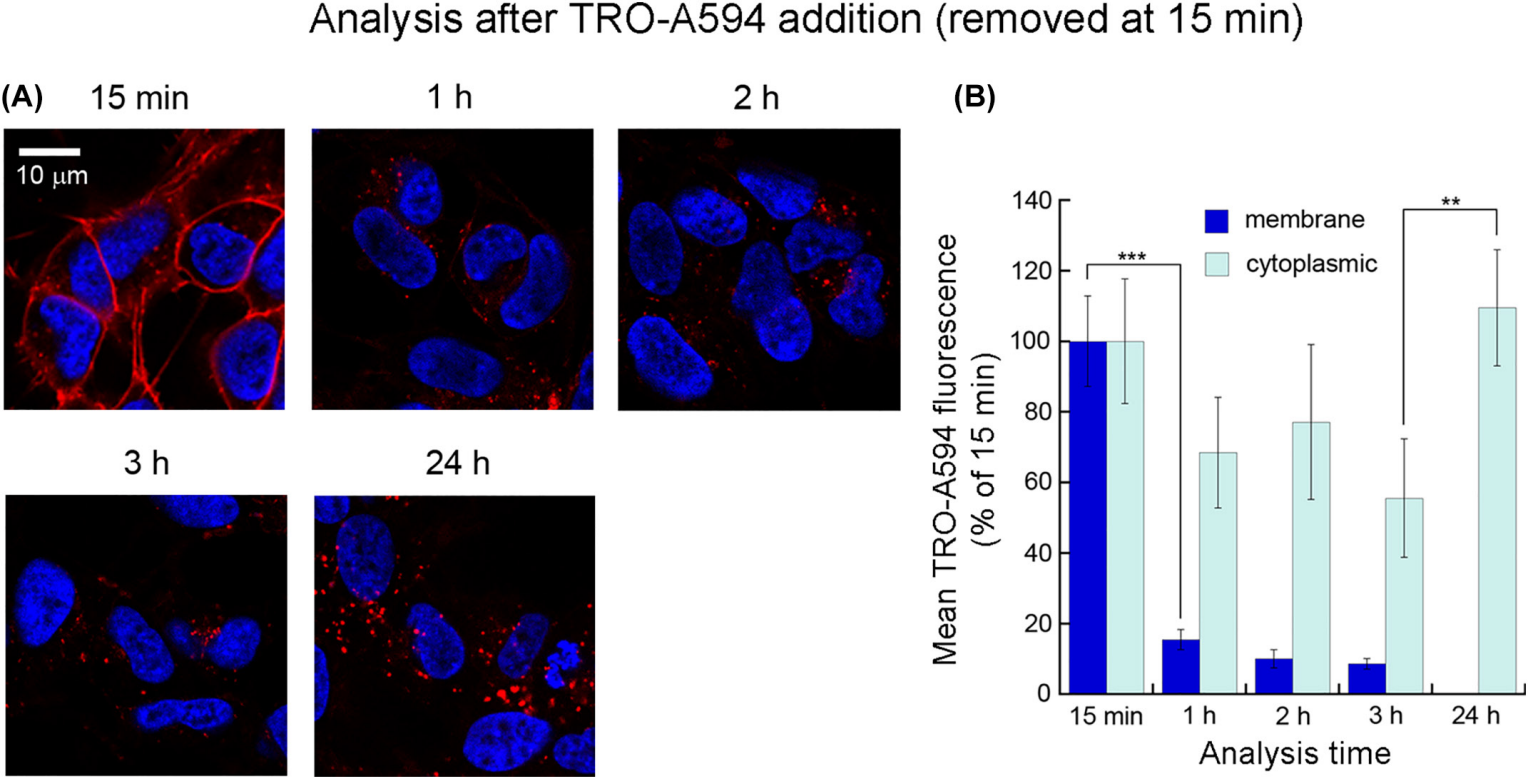
Pulse‐chase of TRO‐A594 in SH‐SY5Y neuroblastoma cells. (A) Representative confocal images of SH‐SY5Y cells incubated with 5 μM TRO‐A594 for 15 min, washed and then imaged at different times after the beginning of incubation. Red, TRO‐A594; blue, Hoechst. The images were analyzed at median planes parallel to the coverslip. (B) Quantitative analysis of plasma membrane (blue bars), and cytoplasmic (light blue bars) trodusquemine‐derived fluorescence. *n* > 50 cells, from three independent experiments; error bars, SD; Student's *t*‐test, **p < .01; ***p < .001 with respect to the immediately preceding incubation time for each compartmental analysis.

In order to follow the internalization pathway of trodusquemine through a pulse‐chase approach, we incubated SH‐SY5Y cells with 5 μM TRO‐A594 for 15 min and then replaced the cell medium with fresh one without trodusquemine to remove the possibly unbound TRO‐A594. Confocal images were acquired immediately after medium replacement (i.e., 15 min) and then at 1, 2, 3, 24, and 48 h (Figure 2A,B). As expected, at 15 min TRO‐A594 localized almost completely to the cell membrane, whereas a very low quantity was present in the cytoplasm. At 1 h, however, we observed a dramatic reduction (more than fivefold) of the aminosterol levels on the plasma membrane, which was not accompanied by a proportional reduction in the cytoplasm. At 2 and 3 h, we did not observe significant changes in TRO‐A594 fluorescence. After 24 h, the TRO‐A594 signal disappeared from the cell membrane, whereas it increased the cytoplasm. Similar values were found at 48 h.

The lack of further reduction after such long times, where a decrease could have been expected following cell division, can be justified by the anti‐proliferative effect of trodusquemine.^40^ The dramatic decrease (~85%) in membrane fluorescence intensity at 1 h may be due to the equilibrium between the membrane‐bound and ‐unbound states with consequent removal of TRO‐A594 from the medium. Compared to unlabeled trodusquemine, which is known to be fully bound to LUVs at the 5 μM concentration used here,^24^ the hydrophilic nature of the fluorophore is likely to increase its water solubility.

To explore the importance of the chemical nature of the dye, we used TRO‐ATTO565, where the dye is neutral, less hydrophilic and is not, therefore, expected to change the positive charge of trodusquemine. TRO‐ATTO565 displayed a behavior similar to TRO‐A594, as it bound almost completely to the membrane after 15 min with a very low quantity present in the cytoplasm (Figure S4). However, it persisted longer on the cell membrane as a ~ 10% decrease was found at 1 h, ~X% decrease was evident at 2 h and X% decrease at 3 h (Figure S4). This indicated a lower solubility of TRO‐ATTO565 relative to TRO‐A594, with lower membrane‐unbound aminosterol.

The images also show that internalized TRO‐A594 was not diffusely localized in the cytoplasm, but was rather organized in vesicular clusters. While the rapid internalization of trodusquemine on short timescales (15 min) is compatible with macropinocytosis (or phagocytosis) processes, the slower and steady decrease in the membrane levels accompanied by the increase in the cytoplasmic fraction might be ascribed to endocytic events.

### 3.3 Intracellular TRO‐A594 localizes mostly in lysosomes

**FIGURE 3:**
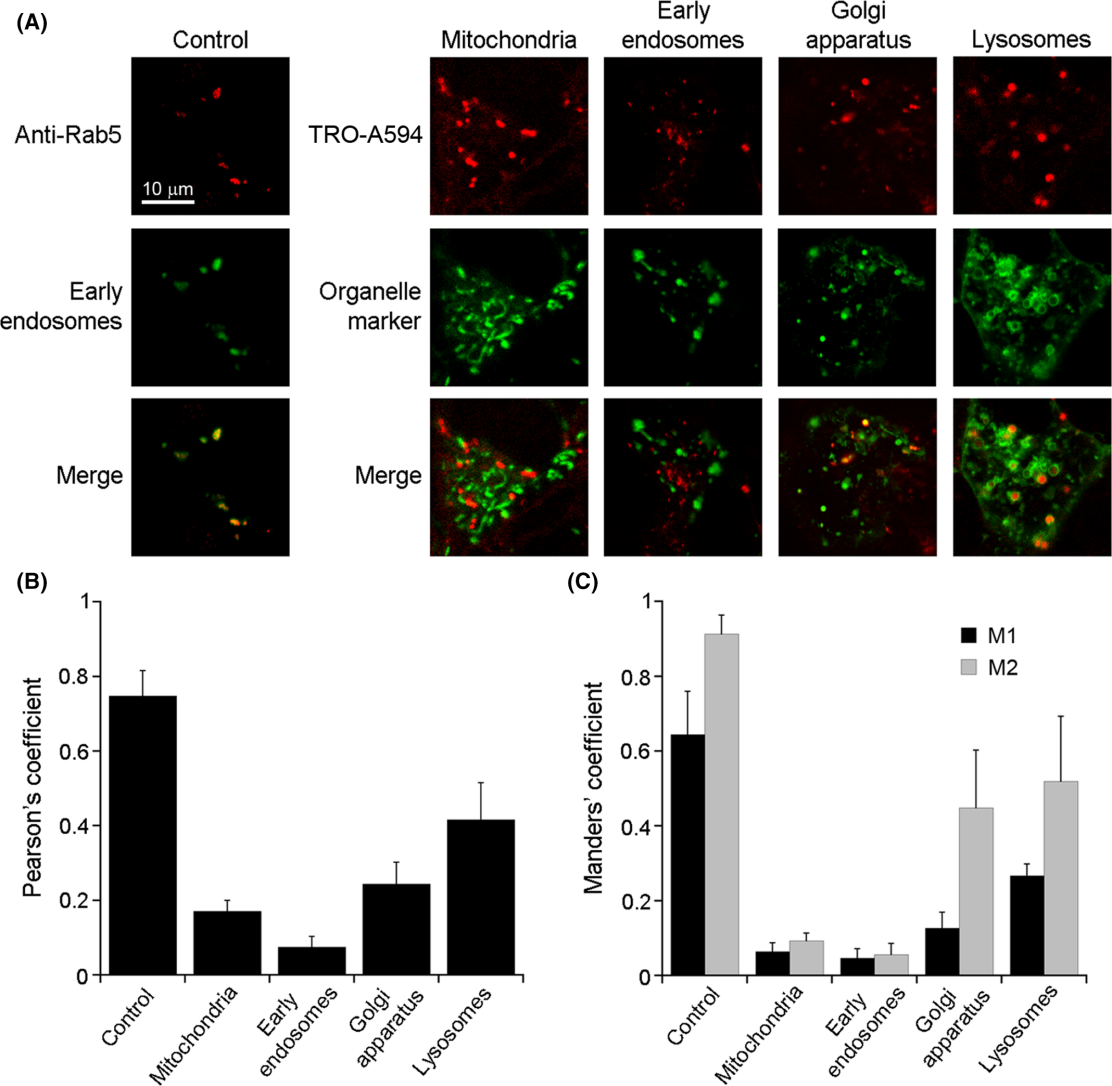
Colocalization of TRO‐A594 with cytoplasmic organelles. (A) Representative confocal images of SH‐SY5Y cells incubated with 5 μM TRO‐A594 for 15 min. The cells were then washed and the confocal acquisitions were made at 2 h. To label mitochondria, cells were treated with MitoTracker™ Green FM for 15 min before confocal acquisition. To label early endosomes, Golgi apparatus and lysosome cells had been transiently transfected 24 h earlier with GFP‐EEA1 wt, mEmerald‐TGNP‐N‐10, and LAMP1‐mGFP plasmids, respectively. As a positive control, cells overexpressing the GFP‐EEA1 were fixed and immunolabeled with anti‐Rab5 antibody coupled to secondary Alexa 568 antibody. (B and C) Histograms reporting (B) the mean cell Pearson's and (C) the Manders' colocalization coefficients (M1, fraction of subcellular compartment overlapping with TRO‐A594; M2, fraction of TRO‐A594 overlapping with the subcellular compartment). The analysis was performed at median planes of 40–45 cells after subtracting background, in two different experiments. Error bars, SD.

In order to characterize the nature of the observed vesicular clusters, we looked at the colocalization of TRO‐A594 with markers of various cytoplasmic organelles 2 h after incubation and washing at 15 min (Figure 3A–C). Confocal images showed an almost complete absence of colocalization between TRO‐A594 and both MITOtracker and GFP‐EEA1, used to label mitochondria and early endosomes, respectively. A significant, yet very low degree of colocalization, was observed for TRO‐A594 and the Golgi apparatus, labeled with mEmerald‐TGNP‐N‐10. By contrast, a higher degree of colocalization was measured for TRO‐A594 and lysosomes, marked using LAMP1‐mGFP. It is important to note that most of the intracellular fluorescence signal of TRO‐A594 appeared to be in part situated in the lumen of LAMP1‐mGFP positive vesicles (Figure 3A and Movie S1).

The highest Pearson's coefficient value was obtained for the colocalization of TRO‐A594 and lysosomes (0.416 ± 0.099), followed by the colocalization with Golgi apparatus (0.244 ± 0.058), mitochondria (0.171 ± 0.029), and early endosomes (0.075 ± 0.029) (Figure 3B). As a positive control of high degree of colocalization, the subcellular targeting of Rab5, a key regulator of endosome biogenesis and trafficking^41^, ^42^ was analyzed in cells overexpressing the early endosome marker GFP‐EEA1, leading to a Pearson's coefficient value of 0.748 ± 0.068. The Manders' coefficients, indicated as M1 (fraction of subcellular compartment overlapping with trodusquemine) and M2 (fraction of TRO‐A594 overlapping with the subcellular compartment), reflected with good approximation the Pearson's coefficient values (Figure 3C). In general, we observed rather low M1 and M2 values in the cases of colocalization with mitochondria (M1 = 0.064 ± 0.023; M2 = 0.093 ± 0.020) and early endosomes (M1 = 0.046 ± 0.025; M2 = 0.056 ± 0.029), whereas the values were higher for the colocalization with Golgi apparatus (M1 = 0.126 ± 0.042; M2 = 0.448 ± 0.153) and, even more, with lysosomes (M1 = 0.267 ± 0.031; M2 = 0.519 ± 0.174). The positive control led to high M1 and M2 values (M1 = 0.644 ± 0.115; M2 = 0.913 ± 0.050). To check if these results were affected by the acquisition time, the same experiments and colocalization analysis were also performed at 24 h. The Pearson's and Manders' coefficient values (Figure S5A,B) were comparable to those obtained for the samples acquired at 2 h (Figure 3B), indicating that the definitive subcellular targeting of TRO‐A594 was reached at a very early stage.

Pearson's and Manders' coefficients for estimating the colocalization of TRO‐A594 and lysosomes were not found as high as for the Rab5/GFP‐EEA1 control since the aminosterol signal was found in part in the interior of the vesicles (Figure 3A).

A similar co‐localization pattern with lysosomes was also found for TRO‐ATTO565, confirming again that the behavior of trodusquemine is not affected by the conjugated dye (Figure S5C). Moreover, to exclude artifacts due to the overexpression of LAMP1‐mGFP, we confirmed the co‐localization with lysosomes using the lysosomal marker LysoView™ 488 (Figure S5D).

### 3.4 Lower binding of TRO‐A594 to cholesterol‐depleted cells

**FIGURE 4:**
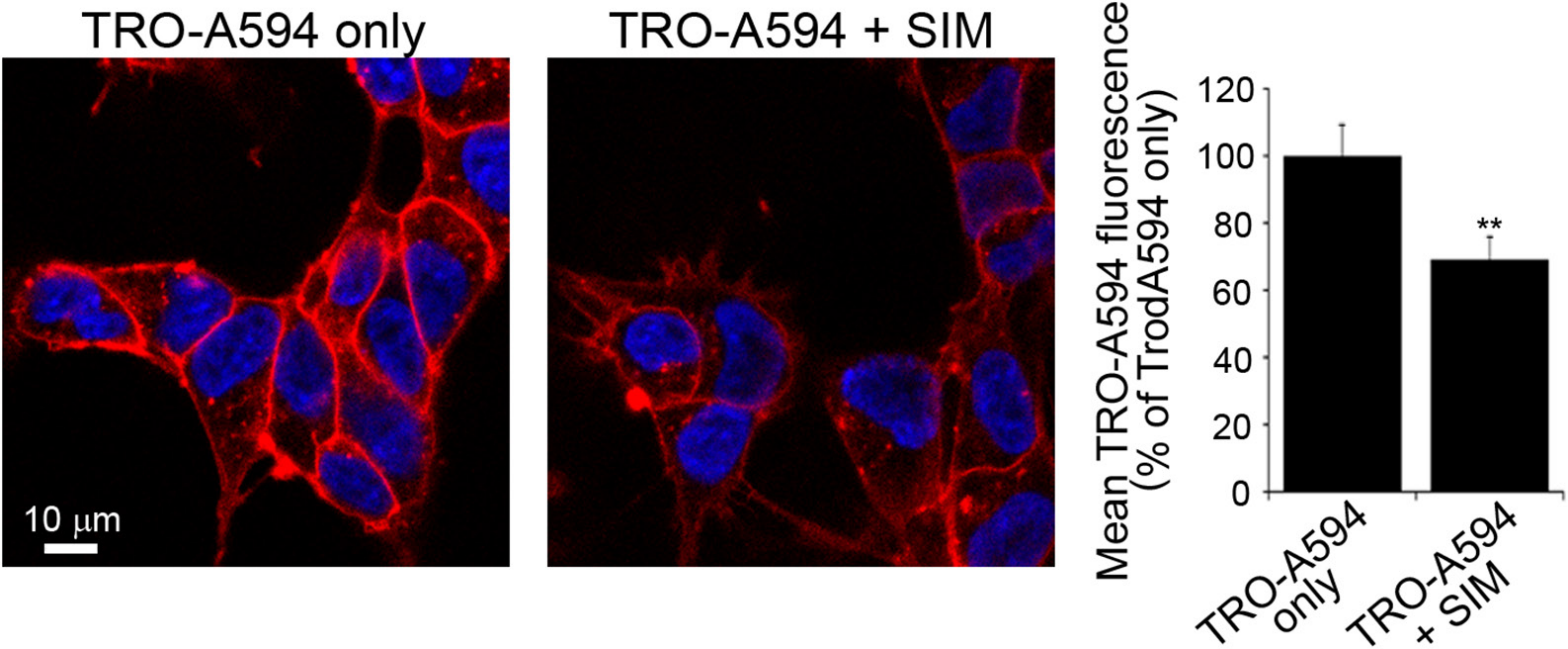
Effect of cholesterol depletion on TRO‐A594 binding affinity. Representative confocal images of SH‐SY5Y cells treated for 48 h with or without 10 μM Simvastatin (SIM), and then incubated with 5 μM TRO‐A594 for 15 min before confocal image acquisition. Blue and red fluorescence intensities indicate nuclei labeled with the dye Hoechst and trodusquemine, respectively. The images were analyzed at median planes parallel to the coverslip. *n* > 50 cells, from three independent experiments; error bars, SD; Student's *t*‐test, **p < .01 with respect to TRO‐A594 only.

FRET experiments in LUVs have demonstrated that TRO‐BODIPY TMR‐X has a good affinity for cholesterol, higher than that observed for GM1 and sphingomyelin.^24^ To provide additional evidence in vivo, neuroblastoma cells were treated with Simvastatin, a member of the statin drug class well known to inhibit the pathway leading to cholesterol production,^43^, ^44^ for 48 h, followed by the treatment with 5 μM TRO‐A594 for 15 min. Confocal imaging and analysis revealed that in cholesterol‐depleted cells the binding degree of TRO‐A594 was reduced by ca. 30% with respect to cells treated with TRO‐A594 only (Figure 4), providing further evidence on the relevant role of cholesterol in the binding affinity of trodusquemine to the cell membrane.

### 3.5 TRO‐A594 and TRO‐ATTO565 display high affinity to fixed mouse nerve fibers

**FIGURE 5:**
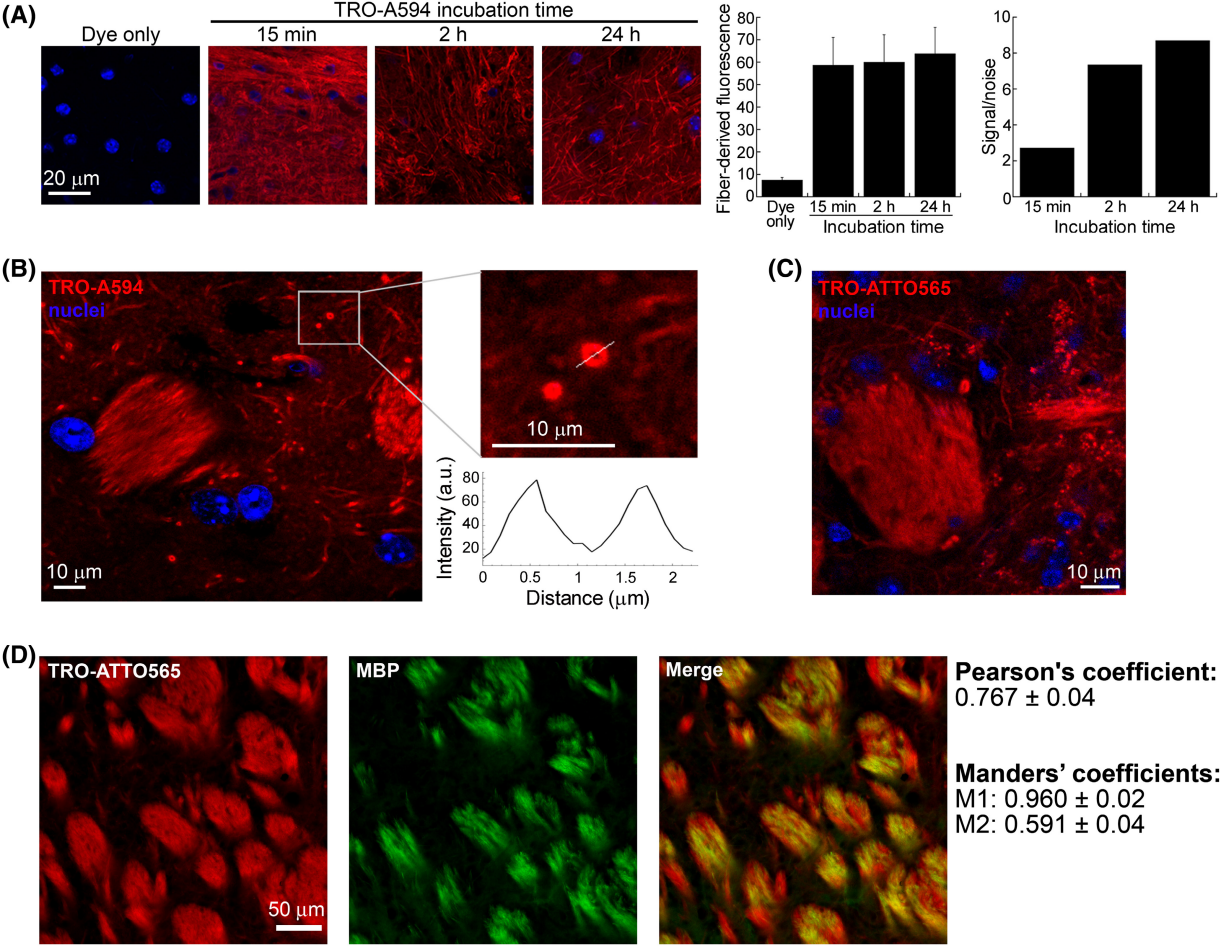
TRO‐A594 as marker of mouse nerve fibers. (A) Representative confocal images of fixed mouse brain slices treated with 0.5 μM Alexa Fluor® 594 (dye only, corresponding to the concentration used in 5 μM TRO‐A594) and 5 μM TRO‐A594 for 15 min, 2, and 24 h. The histograms show the quantitative values of TRO‐A594 fluorescence associated with nerve fibers (left) and the signal‐to‐noise ratio (right). Error bars, SD. Objective lens 60×/NA 1.4; excitation light 405 and 561 nm. (B) Confocal image of TRO‐A594‐stained fiber bundles and single fibers after 24 h incubation. The magnified image shows a sectioned fiber stained with TRO‐A594. The graph reports the intensity fluorescence analysis of the sectioned fiber. Objective lens 60×/NA 1.4; excitation light 405 and 561 nm. (C and D) Confocal images of (C) TRO‐ATTO565‐stained fiber bundles and single fibers (red) and (D) their overlay with anti‐MBP and secondary Alexa 488 staining (green). A colocalization analysis of TRO‐ATTO565 with MBP was carried out and the mean Pearson's and Manders' colocalization coefficients (M1, fraction of MBP overlapping with TRO‐ATTO565; M2, fraction of TRO‐ATTO565 overlapping with MBP) ± SD are reported in the figure.

It is known that the dry mass of myelin, that is, the sheath that covers nerve fibers, is mainly composed of lipids (ca. 70%), and among them cholesterol is one of the most represented.^45^ Confocal microscopy imaging was then performed on mouse brain samples in order to investigate the ability of TRO‐A594 to interact with nerve fibers in 100‐μm thick mouse slices. TRO‐A594 at the same concentration used for all the experiments reported above (5 μM) was incubated for 15 min, 2, and 24 h (Figure 5A). Already after 15 min, TRO‐A594 appeared distributed through the whole slice, showing a particularly high affinity for the nerve fibers. The fluorescence intensity did not change significantly at longer incubation times (2 and 24 h), even after several washing steps, indicating a very tight and selective association between trodusquemine and nerve fibers. As a control, slices were also incubated for 15 min with 0.5 μM Alexa Fluor® 594 dye in the absence of trodusquemine, in order to exclude that dye could per se bind to the sample. The absence of fiber staining, and the fact that also TRO‐ATTO565 showed the same type of labeling pattern (Figure 5C), confirmed that the specific nerve labeling in the presence of TRO‐A594 was entirely attributable to the aminosterol. The increase in the signal‐to‐noise ratio as a function of the incubation time (passing from 2.7 after 15 min, to 7.3 after 2 h and 8.8 after 24 h) (Figure 5A), the detection of the typical disposition of fiber bundles with different orientation^27^ (Figure 5B) and the overlay with MBP staining (Figure 5D) demonstrate the selective affinity of labeled trodusquemine for nerve fibers.

**FIGURE 6:**
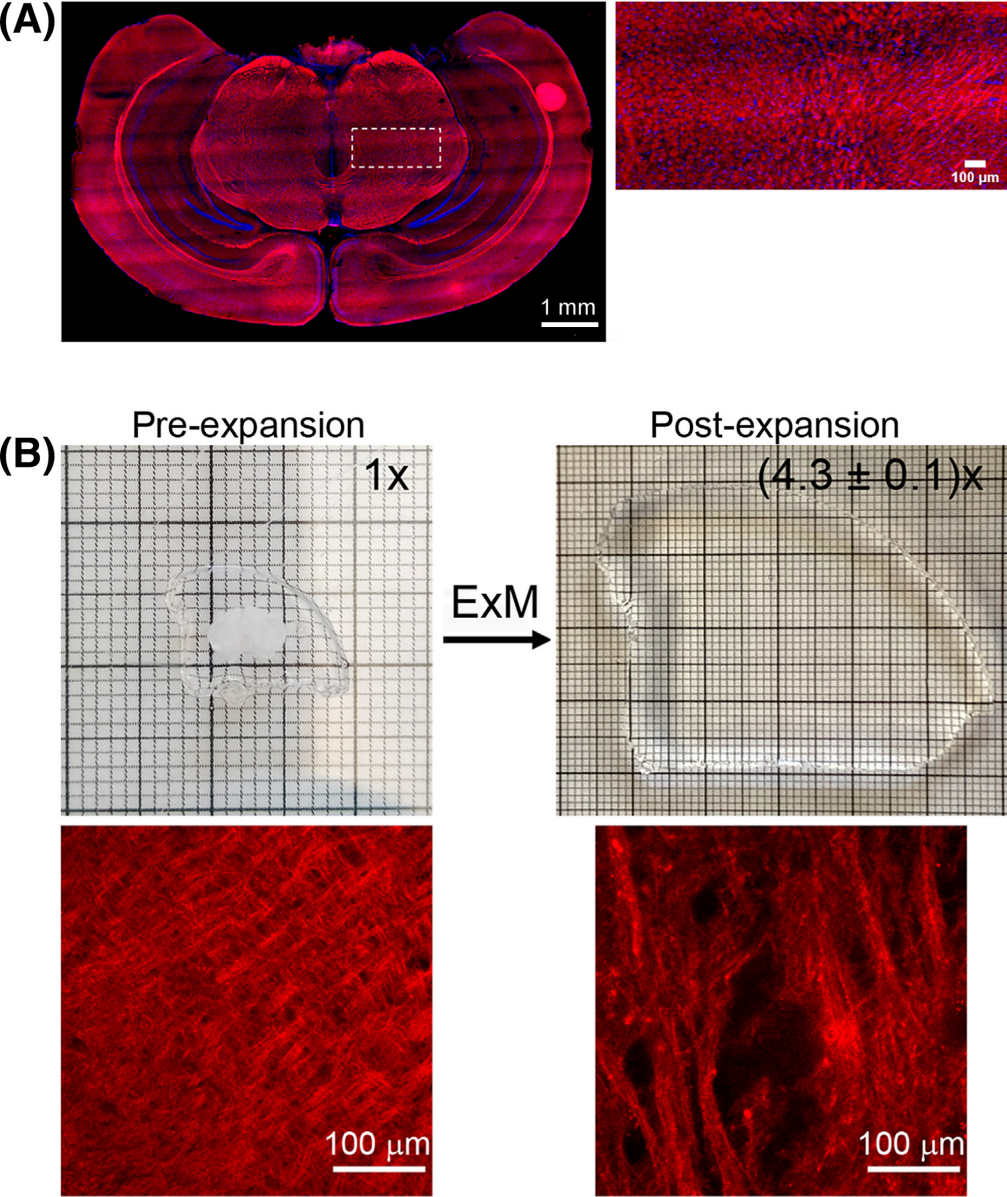
Compatibility of TRO‐A594 with light sheet and expansion microscopy. (A) LSFM downsampled reconstruction of 10‐μm thick mouse brain slice treated with 5 μM TRO‐A594 for 24 h. The magnified image shows the different orientation of fiber bundles labeled with TRO‐A594. Objective lens 12×, NA 0.53; excitation light 405 and 568 nm. (B) Confocal images of pre‐ and post‐expansion mouse brain slices treated with 5 μM TRO‐A594 for 24 h, and the corresponding hydrogels with the expansion factor quantification. Objective lens 20×, NA 0.9; excitation light 405 and 568 nm.

LSFM was then used to map TRO‐A594 in a whole mouse cleared brain section (Figure 6A). After treatment with TRO‐A594, the specimen was equilibrated with increasing percentages of mixture of TDE/PBS solutions (see M&M). The compatibility of TRO‐A594 with such clearing agent was ascertained by imaging the treated slices on confocal microscope after 24 h with 5 μM TRO‐A594 and upon exchanging PBS with TDE, and vice versa; the fluorescent signal of TRO‐A594 was not affected by the type of maintaining solution and its binding to the nerve structures was retained (Figure S6). The images obtained with confocal microscopy (Figure S6) and the whole‐slice reconstruction by LSFM (Figure 6A) demonstrate the high affinity for the nerve fiber and the fluorescence retention of TRO‐A594 upon the TDE treatment.

Finally, we tested the compatibility of TRO‐A594 as a nerve marker with ExM to fully characterize its signal retention using an additional clearing protocol. Although ExM is a super‐resolution approach, it can also be considered as a clearing method since, at the final expansion, the tissue is 99% water. For these experiments, the fixed samples were treated with 5 μM TRO‐A594 for 24 h and then incubated with the succinimidyl ester AcX, which can react with the secondary amines of trodusquemine (most of the primary amines group of the proteins have already reacted with PFA) to yield acrylamides that can then be copolymerized into the subsequent polyacrylamide gel matrix, in which fractured regions were not detected, indicative of an isotropic expansion after the digestion step (Figure 6B). The ExM approach was also successfully tested in cells that were first incubated with TRO‐A594 and then fixed (Figure S7).

## 4 DISCUSSION

Although aminosterols such as squalamine and trodusquemine have been known for more than 20 years, and have been largely used to treat different pathological conditions, their trafficking in the cell is still a matter of investigation. Here, we have incubated human neuroblastoma cells with trodusquemine coupled to fluorophores having different degrees of charge and hydrophilicity, and studied the dynamics and features of its binding to the plasma membrane and subsequent internalization. By using fluorophores that could change the overall positive net charge of trodusquemine (Alexa Fluor® 594) or leave it unaffected (ATTO 565), which was found to be important for some specific biological features,^7^, ^46^ we demonstrated that altering this physicochemical parameter influences only marginally the binding of trodusquemine to the plasma membrane and its trafficking, encouraging the use of fluorescent probes to follow the cellular localization of trodusquemine and aminosterols. In particular, it was found that TRO‐ATTO565 binds to the membrane more tightly, reducing the membrane‐unbound probe. This causes the molecule to remain on the membrane longer and be internalized to a larger amount relative to TRO‐A594.

The fact that we could initially detect trodusquemine on the plasma membrane of a cell is coherent with the idea that it can displace the binding of Aβ oligomers from the surface of the cell.^14^, ^22^, ^23^, ^47^, ^48^ We found that the interaction of TRO‐A594 with the plasma membrane seems to be characterized by a dynamic equilibrium between the membrane‐bound and unbound states, in contrast with the finding that unlabeled trodusquemine partition entirely on a LUV lipid bilayer.^24^ This behavior could be in part explained by the additional hydrophilic moiety introduced by Alexa Fluor® 594. Indeed, labeling with the less hydrophilic and charged ATTO 565 probe reduces the membrane‐unbound pool of trodusquemine and shifts the equilibrium toward the membrane‐bound state. According to our results, a fraction of TRO‐A594 present initially in the cell medium can be internalized within minutes via macropinocytosis, while the fraction of TRO‐A594 remaining bound to the plasma membrane after replacing the medium is gradually cleared and internalized in the subsequent hours, probably through standard endocytic events taking place in the cell. A similar remark can be applied to explain the behavior of TRO‐ATTO565, with the only difference that its clearance from plasma membrane is slower compared to that of TRO‐A594. In light of the amphipathic nature of trodusquemine, it is intriguing to note that macropinocytosis, although being a not‐selective process of uptake, can be facilitated by the right balance of high hydrophobicity and positive net charge of the molecules.^49^, ^50^ The higher positive net charge and higher hydrophobicity of the unlabeled trodusquemine could lead to increase further macropinocytosis events (in agreement with an increased intracellular uptake observed for TRO‐ATTO565). From a speculative point of view, a potential stimulation of the macropinocytotic/phagocytic pathway by trodusquemine could be functional both for the clearance of extracellular pathogenic organisms and accumulated misfolded/aggregated proteins.

Notably, it was recently found that pharmacological inhibition of PTP1B with trodusquemine prevents hippocampal neuron loss and spatial memory deficits in a transgenic Alzheimer's disease mouse model with Aβ pathology.^47^ In our experiments, most of the intracellular TRO‐A594 was found at the level of the lysosomes and, to a lower extent, at the Golgi level. The localization of trodusquemine at the lysosomal level could be beneficial for restoring the activity of neuronal lysosomes engulfed by aggregated proteins in Alzheimer's and Parkinson's diseases. In addition, our results are in agreement with the hypothesis that PTP1B dephosphorylates the activated insulin receptor intracellularly and with the finding that PTP1B is inhibited by trodusquemine.

In agreement with previous FRET results obtained with LUVs showing a higher binding preference of TRO‐BODIPY TMR X, used as an acceptor, and cholesterol‐BODIPY‐FL, used as a donor,^24^ we found that cholesterol depletion in cells reduces the affinity of TRO‐A594 for the cell membrane. We also discovered that TRO‐A594 displays a very high affinity toward myelinated nerve fibers, which are in fact enriched with cholesterol. Moreover, the emerging evidence that the lipid‐rich myelin sheath is impaired by the presence of neuritic plaques formed by the Aβ accumulation^51^, ^52^, ^53^ suggests that a potential treatment with trodusquemine may help in protecting the sheath integrity from the action of Aβ plaques.

In addition to exploring the biology of an aminosterol, our experiments have highlighted several innovative applications of TRO‐A594 at the methodological level. Interestingly, TRO‐A594 has been recently used as a generic marker for the plasma membrane.^24^, ^54^ Here, we demonstrated the suitability of TRO‐A594 for two advanced microscopy approaches: LSFM and ExM. LSFM was used to map TRO‐A594 in a whole mouse brain slice. For improving the imaging of molecular probes and performing deep tissue imaging, optical techniques require high transparency and optical free‐aberration samples.^55^ TDE is one of the most common, quick, and inexpensive clearing agents,^26^, ^27^ which was efficiently combined with TRO‐A594 (or TRO‐ATTO565) in mouse slices, showing an efficient fluorescence signal retention upon treatment. The preservation of its fluorescence signal in TDE‐equilibrated samples suggests that its use as a neuronal marker may be applied to different experimental approaches. On the other hand, ExM uses a swellable polyelectrolyte hydrogel which is crosslinked throughout the specimen to physically expand it fourfold and obtain an optical resolution of 70 nm using confocal microscopy.^28^, ^56^ Up to now, this technique has been mainly used to investigate macromolecular complexes by crosslinking proteins to the hydrogel, demonstrating a uniform expansion process with an accuracy of about 20 nm.^57^ Here, we show that ExM can be successfully applied to mouse brain slices and cultured neuroblastoma cells treated with TRO‐A594. Although the mechanism is not clear at the moment, we found that the TRO‐A594 fluorescence is retained during the expansion process using commercially available handles (MA‐NHS and AcX),^28^ most probably via crosslinking the free amines of TRO‐A594 to the hydrogel. It is known that PFA, GA, and N‐hydroxy succinimide (NHS) esters crosslinkers have a higher preference for primary amines^58^ but are not exclusive for them and can cross link other groups including secondary amines^59^, ^60^ (most aldehydes and ketones also react with 2°‐amines to give products known as enamines). In addition, other lipophilic dyes have been found to be compatible with the CLARITY clearing technique, for some of which the occurring chemical reactions are still unknown.^61^ Although several phospholipid membrane^62^, ^63^ and fiber stainings have been developed for labeling clarified tissues,^64^, ^65^ here we demonstrated the compatibility of our labeled trodusquemine with two different mouse brain clearing approaches, TDE and ExM. These results demonstrate that TRO‐A594 has a particularly high affinity for nerve fibers and can therefore be used as a generic post‐fixation marker for the white matter, suitable for advanced microscopy techniques such as LSFM and ExM.

## AUTHOR CONTRIBUTIONS

Claudia Capitini, Luca Pesce, and Martino Calamai conceived and designed the research; Claudia Capitini, Luca Pesce, Alessandra Franceschini, Giulia Fani, Giacomo Mazzamuto, Massimo Genovese performed the research and acquired the data; Claudia Capitini, Luca Pesce, Martino Calamai, Giacomo Mazzamuto, Giuseppe Pieraccini, Paolo Paoli, Michael Zasloff, and Fabrizio Chiti analyzed and interpreted the data; Martino Calamai, and Francesco S. Pavone supervised the research; Claudia Capitini, Luca Pesce, and Martino Calamai wrote the original draft. All authors were involved in revising the manuscript.

## FUNDING INFORMATION

European Union's Horizon 2020 research and innovation program under grant agreement no. 654148 Laserlab‐Europe, and Horizon 2020 Framework program under grant agreement no. 945539 (Human Brain Project SGA3).

## DISCLOSURES

The authors declare the following competing interests: M.Z. is one of the inventors in a patent for the use of trodusquemine in the treatment of Parkinson's disease. All other authors declare they have no competing interests.

## Supporting information


Appendix S1

Video S1

## Data Availability

The data that support the findings of this study are available on request from the corresponding author. The data are in TIF and Kaleidagraph formats.
