# Supplementary material for: Studying the trafficking of labeled trodusquemine and its application as nerve marker for light‐sheet and expansion microscopy
Source: FASEB J. 2022 Nov 24;36(12):e22655. doi: 10.1096/fj.202201276R (PMC9827910; doi:10.1096/fj.202201276R)
Supplement: Supplementary file 1 — Appendix S1 [file FSB2-36-0-s001.docx]

**Supplementary Information for**

Studying the trafficking of labeled trodusquemine and its application as nerve marker for light-sheet and expansion microscopy

Claudia Capitini, Luca Pesce, Giulia Fani, Giacomo Mazzamuto, Massimo Genovese, Alessandra Franceschini, Paolo Paoli, Giuseppe Pieraccini, Michael Zasloff, Fabrizio Chiti, Francesco S. Pavone, Martino Calamai

**Other supplementary materials for this manuscript include the following:** Movie S1


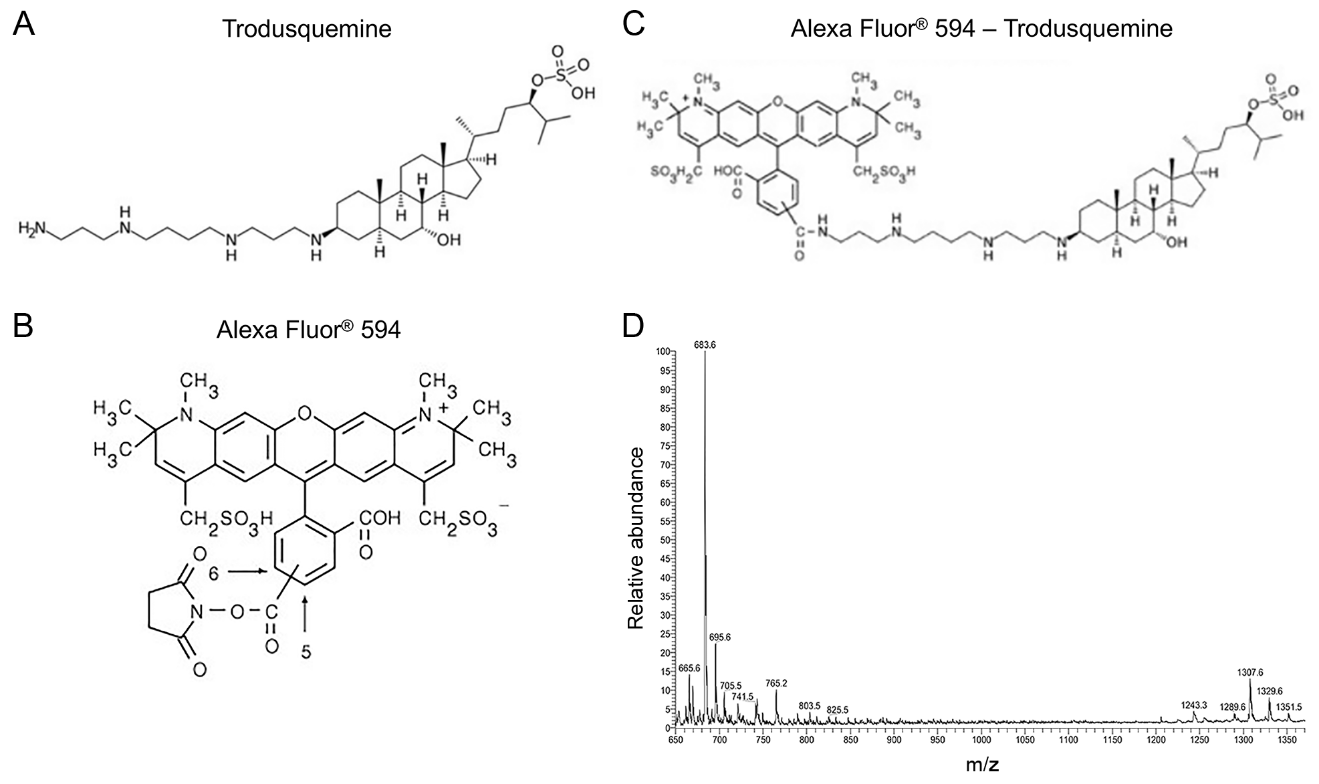


**Figure S1.** (**A**) Structure of trodusquemine. It is formed by a sulfate group (right), a sterol (centre) and a spermine polyamine (left). (**B**) Structure of the dye Alexa Fluor^®^ 594 NHS ESTER used to label primary amines. (**C**) Structure of trodusquemine labeled with Alexa Fluor^®^ 594 NHS ESTER at the level of its terminal primary amine (TRO-A594). Such a dye was used in place of BODIPY TMR-X (1) since it showed a higher solubility degree, and it was more suitable for the excitation wavelengths available on our microscopy set up. Overall, TRO-A594 has a net charge of 0 (**D**) Negative ion electrospray mass spectrum of trodusquemine labeled with Alexa Fluor^®^ 594 NHS ESTER with a molar ratio of 1:10 (dye:trodusquemine), showing the peaks corresponding to unlabeled trodusquemine (m/z 683.6) and labeled-trodusquemine (m/z 1307.6).

**
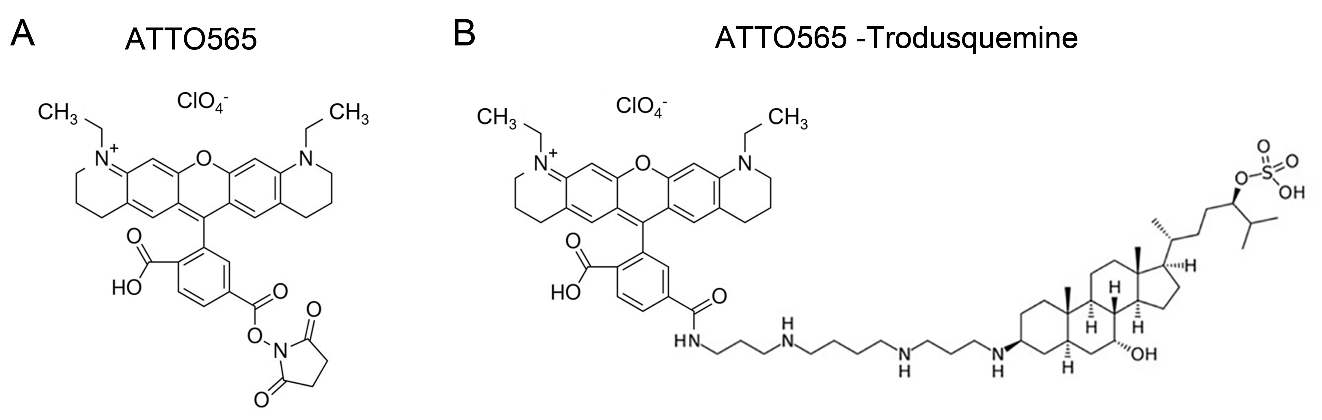
**

**Figure S2.** (**A**) Structure of the dye ATTO 565 NHS ESTER used to label primary amines. (**B**) Structure of trodusquemine labeled with ATTO 565 NHS ESTER at the level of its terminal primary amine (TRO-ATTO565).


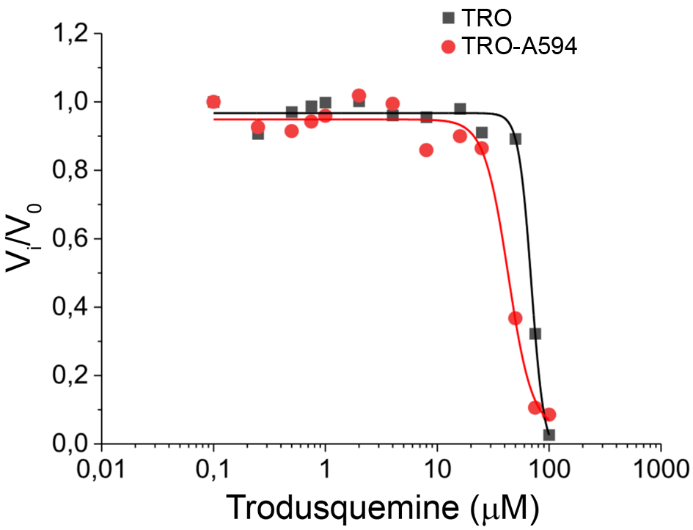


**Figure S3.** Plots reporting the IC_50_ related to the inhibitory activity of TRO and TRO-A594 against PTP1B_1-302_. To measure potential reductions in trodusquemine activity due to the presence of the dye, we evaluated the capability of TRO-A594 to inhibit PTP1B activity *in vitro* by using a truncated form of the human recombinant enzyme and *p*-nitrophenyl phosphate as substrate (33,34), and we compared it with the inhibitory activity exerted by unlabeled trodusquemine (TRO). The IC_50_ values obtained were 43 ± 3.1 µM and 68.7 ± 1.5 µM for TRO-A594 and TRO, respectively, indicating that the labeled aminosterol preserves, even slightly increasing, the inhibitory function against PTP1B.


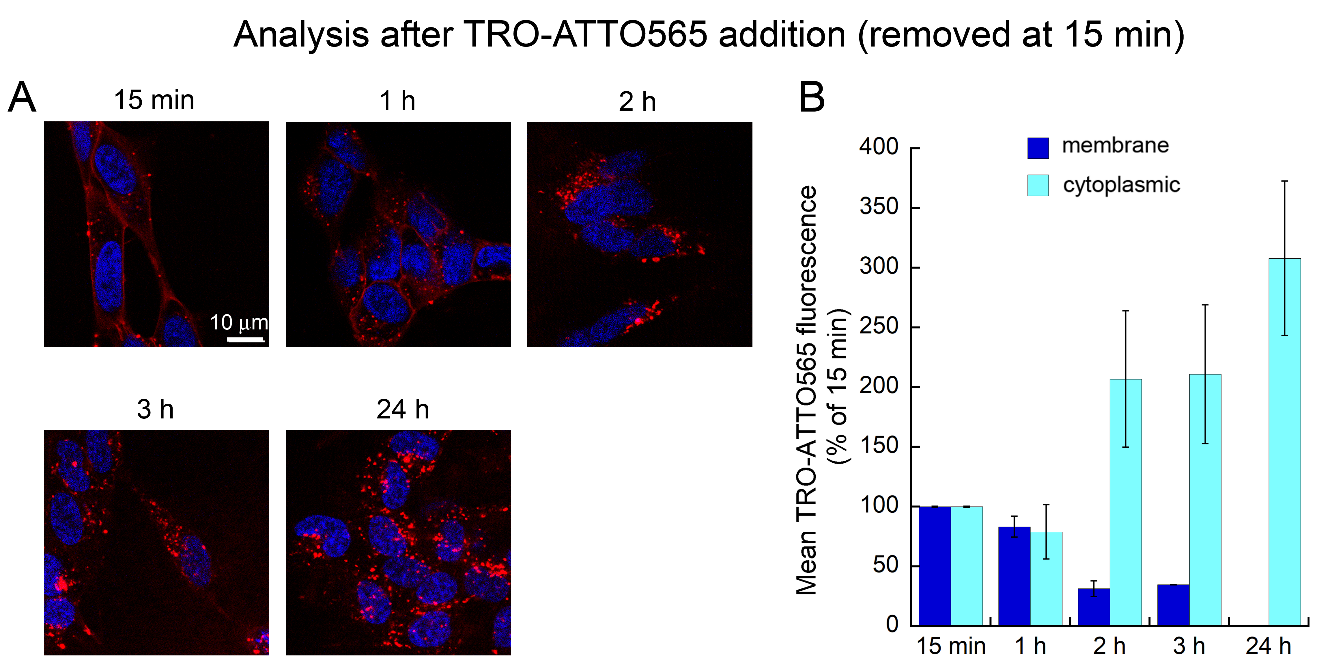


**Figure S4. Pulse-chase of TRO-ATTO565 in SH-SY5Y neuroblastoma cells. (A)** Representative confocal images of SH-SY5Y cells incubated with 5 μM TRO-ATTO565 for 15 min, washed and then imaged at different times after the beginning of incubation. The images were analyzed at median planes parallel to the coverslip. **(B)** Quantitative analysis of plasma membrane (blue bars), and cytoplasmic (light blue bars) trodusquemine-derived fluorescence. n > 50 cells, from 3 independent experiments; error bars, S.D.


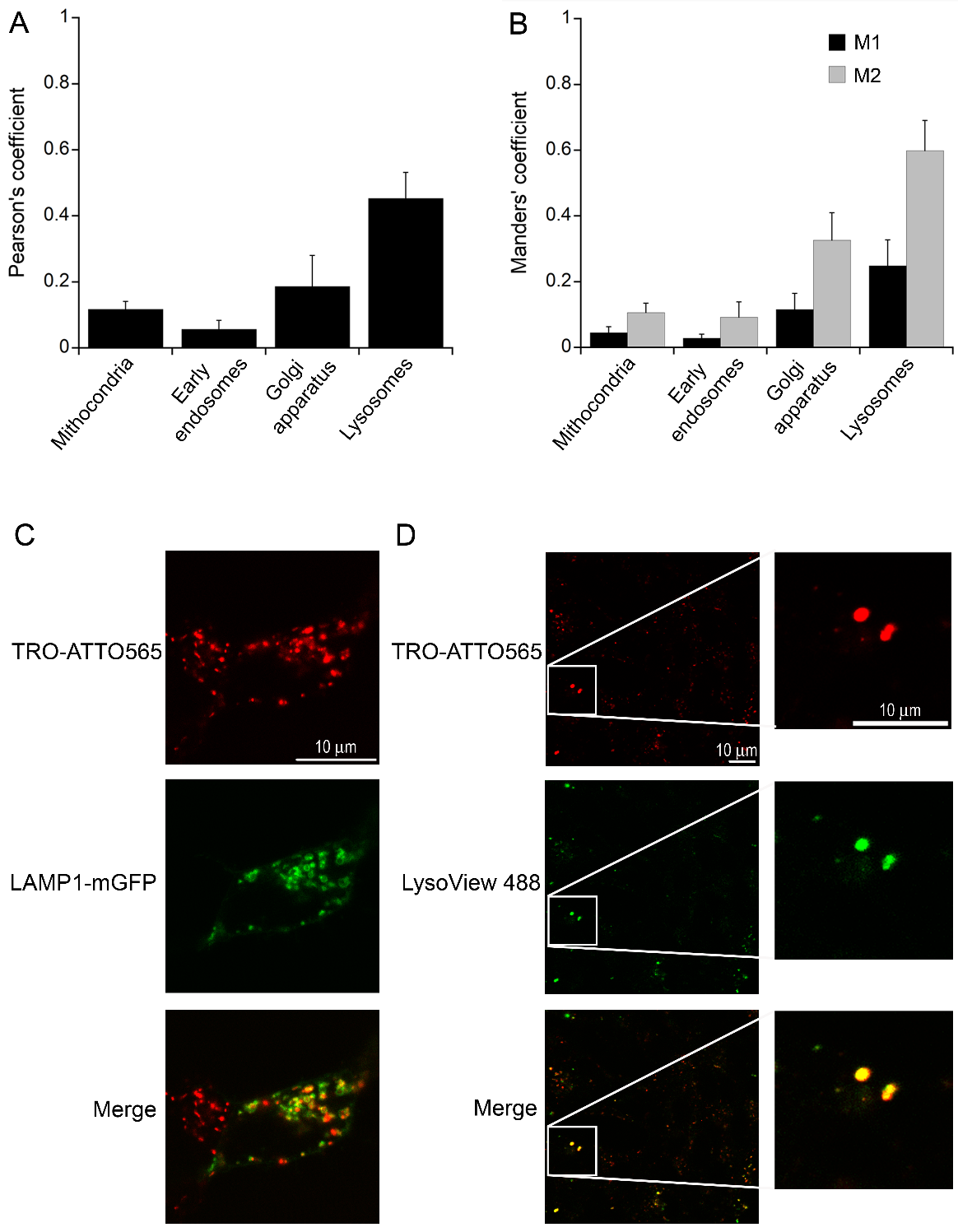


**Figure S5.** Histograms reporting (**A**) the mean cell Pearson’s and (**B**) the Manders’ colocalization coefficients (M1, fraction of subcellular compartment overlapping with TRO-A594; M2, fraction of TRO-A594 overlapping with the subcellular compartment) determined by confocal images of SH-SY5Y cells acquired after 24 h from 15 min-incubation with 5 μM TRO-A594. The analysis was performed at median planes of 40-45 cells after subtracting background, in 2 different experiments. Error bars, S.D. (**C**,**D**) Representative confocal images of SH-SY5Y cells showing the colocalization between TRO-ATTO565 and lysosomes. Cells were incubated with 5 μM TRO-ATTO565 for 2 h. Lysosomes were labelled by transiently transfecting cells 24 h earlier with LAMP1-mGFP plasmid (**C**) or by incubating cells with the LysoView^TM^ 488 dye for 30 min before confocal acquisition (**D**). The Pearson’s coefficients were 0.544 ± 0.13 and 0.543 ± 0.18, while the Manders’ coefficients were M1 = 0.586 ± 0.18, M2 = 0.323 ± 0.16 and M1 = 0.391 ± 0.13, M2 = 0.662 ± 0.22, for colocalization with LAMP1-mGFP and LysoView^TM^, respectively.

**
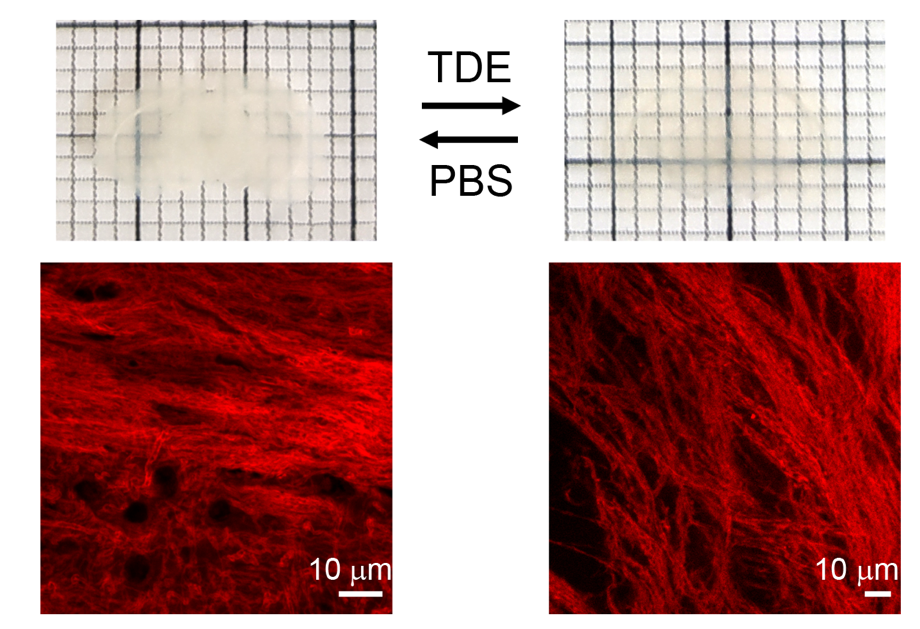
**

**Figure S6.** Analysis of the compatibility of TRO-A594 with the clearing agent TDE in mouse brain. Representative confocal images of mouse brain sections treated with 5 μM TRO-A594 for 24 h and equilibrated exchanging PBS with TDE, and vice versa.

**
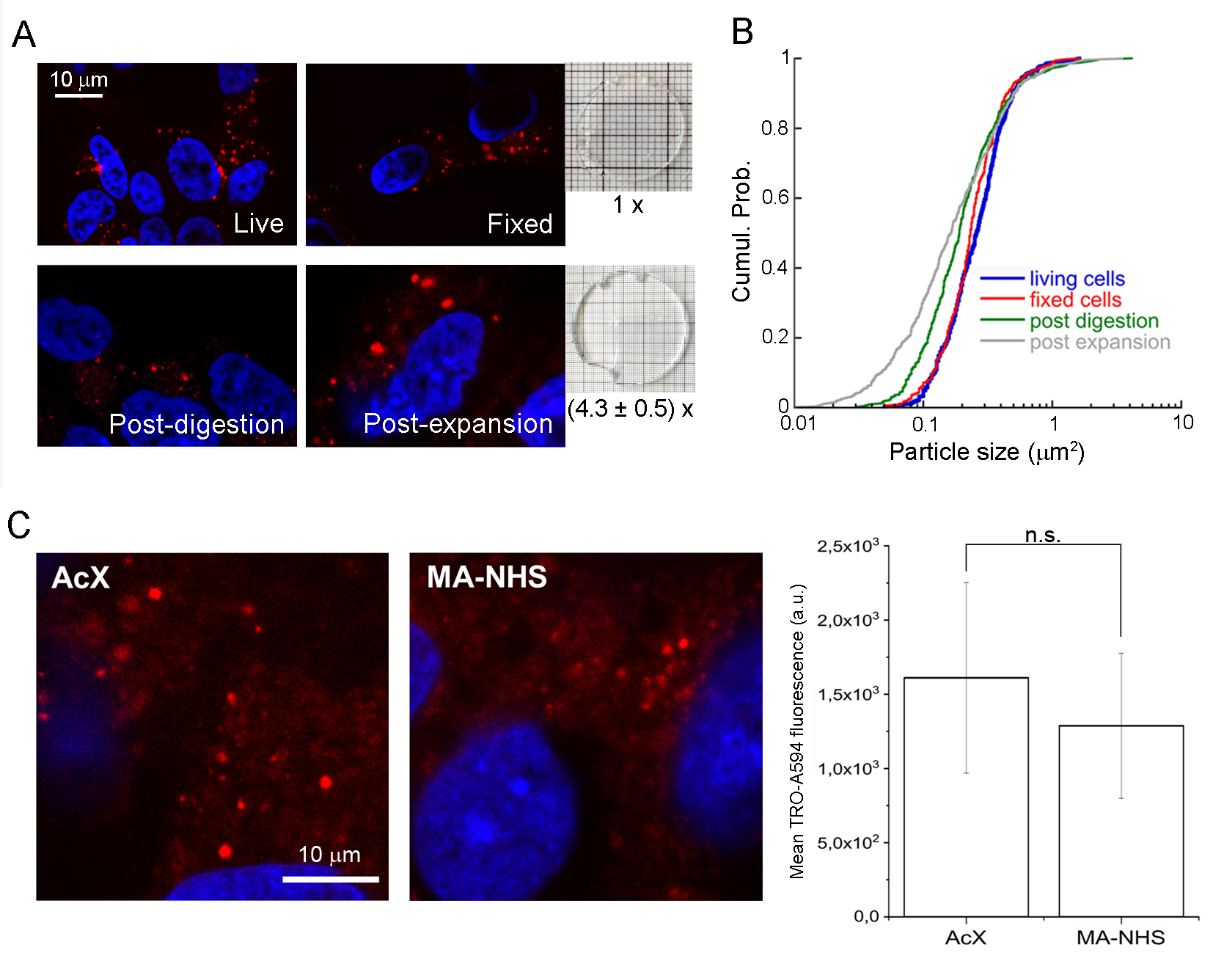
**

**Figure S7. ExM of TRO-A594 labeled cells.** The ExM technique allows the resolution limit of the conventional light microscopy to be overcome by physically expanding the biological sample (2, 3). Although such a method was developed for achieving nanoscale information of labeled proteins and nucleic acids, we have proved its compatibility with a lipid like trodusquemine in which amines are present. Live and fixed cell images showed a similar distribution and morphology of the TRO-A594 clusters in the cytoplasm (**A**). Such a result was corroborated by the particle size analysis of the TRO-A594 vesicles in both types of samples, as indicated by the good overlap between the corresponding size distributions (**B**). We quantified and compared the particle size of the TRO-A594 vesicles in pre- and post- expanded samples. The good correlation between the macroscale and nanoscale expansion factor allows us to normalize the particle size with the magnified factor of the hydrogel (4).The size distribution of such spots progressively shifted towards lower size values passing from live/fixed cells (EF = 1) to post-digestion samples (EF = 1.8 ± 0.3) and post-expansion samples (EF = 4.3 ± 0.5), indicating that in the expanded samples we could reveal a population of TRO-A594 vesicles of smaller size, which was masked before because of the limit of diffraction. In our experiments, the minimal diameter of the detectable vesicles decreased from 250 nm in pre-expansion to 120 nm in post-expansion samples, indicating at least a two-fold increase in max resolution. Objective lens 60x/NA 1.4; excitation light 488 nm and 561 nm. (**C**) Representative confocal images of SH-SY5Y cells treated with 5 μM TRO-A594 for 24 h and expanded using both AcX and MA-NHS as crosslinking agents. The histogram reports the TRO-A594 fluorescence determined for both samples. The analysis was performed at median planes of 30-35 cells after subtracting background, in 2 different experiments. Error bars, S.D. The crosslinking agents AcX and MA-NHS were both able to retain the fluorescence signal of TRO-A594, although the former provided a higher fluorescence intensity.

**Movie S1 (separate file).** Time-lapse video of living neuroblastoma cells showing TRO-A594-containing LAMP1-mGFP positive vesicles moving through the cytoplasm. TRO-A594 mostly localizes in the lumen of the lysosomes.

**References**

1. Errico, S., Lucchesi, G., Odino, D., Muscat, S., Capitini, C., Bugelli, C., Canale, C., Ferrando, R., Grasso, G., Barbut, D., Calamai, M., Danani, A., Zasloff, M., Relini, A., Caminati, G., Vendruscolo, M., and Chiti, F. (2020) Making biological membrane resistant to the toxicity of misfolded protein oligomers: A lesson from trodusquemine. *Nanoscale* **12**

2. Chen, F., Tillberg, P. W., and Boyden, E. S. (2015) Optical imaging. Expansion microscopy. *Science* **347**

3. Wassie, A. T., Zhao, Y., and Boyden, E. S. (2019) Expansion microscopy: principles and uses in biological research. *Nat. Methods* **16**

4. Pesce, L., Cozzolino, M., Lanzanò, L., Diaspro, A., and Bianchini, P. (2019) Measuring expansion from macro- to nanoscale using NPC as intrinsic reporter. *J. Biophotonics*
